# Screening for venous thromboembolism in patients with COVID-19

**DOI:** 10.1007/s11239-021-02474-8

**Published:** 2021-05-21

**Authors:** Christophe Vandenbriele, Diana A. Gorog

**Affiliations:** 1 Department of Cardiovascular Diseases, University Hospitals Leuven, Leuven, Belgium; 2 Intensive Care Unit, Royal Brompton Hospital, London, UK; 3 Faculty of Medicine, National Heart and Lung Institute, Imperial College, London, UK; 4 School of Life and Medical Sciences, Postgraduate Medical School, University of Hertfordshire, Hertfordshire, UK

## Abstract

Pulmonary thromboembolism and deep venous thrombosis occur frequently in hospitalised patients with COVID-19, the prevalence increases on the intensive care unit (ICU) and is very high in patients on extracorporeal membrane oxygenation (ECMO). We undertook a literature review to assess the usefulness of screening for peripheral venous thrombosis or pulmonary thrombosis in patients admitted with COVID-19. Outside of the ICU setting, D-dimer elevation on presentation or marked increase from baseline should alert the need for doppler ultrasound scan of the lower limbs. In the ICU setting, consideration should be given to *routine* screening with doppler ultrasound, given the high prevalence of thrombosis in this cohort despite standard anticoagulant thromboprophylaxis. However, absence of lower limb thrombosis on ultrasound does not exclude pulmonary venous thrombosis. Screening with CT pulmonary angiography (CTPA) is not justified in patients on the general wards, unless there are clinical features and/or marked elevations in markers of COVID-19-associated coagulopathy. However, the risk of pulmonary embolism or pulmonary thrombosis in ICU patients is very high, especially in patients on ECMO, where studies that employed routine screening for thrombosis with CT scanning have uncovered up to 100% incidence of pulmonary thrombosis despite standard anticoagulant thromboprophylaxis. Therefore, in patients at low bleeding risk and high clinical suspicion of venous thromboembolism, therapeutic anticoagulation should be considered even before screening, Our review highlights the need for increased vigilance for VTE, with a low threshold for doppler ultrasound and CTPA in high risk in-patient cohorts, where clinical features and D-dimer levels may not accurately reflect the occurrence of pulmonary thromboembolism.

## Highlights

- The frequency of pulmonary and venous thromboembolism is high in hospitalised patients with COVID-19, with even higher prevalence amongst those on the intensive care unit (ICU) and especially those on extracorporeal membrane oxygenation (ECMO).
- In the ICU setting, consideration should be given to *routine* screening with doppler ultrasound, given the high prevalence of thrombosis despite standard anticoagulant thromboprophylaxis. In patients on ECMO, studies employing routine screening with CT scanning have uncovered up to 100% incidence of pulmonary thrombosis despite anticoagulant thromboprophylaxis. Absence of lower limb thrombosis on ultrasound does not exclude pulmonary venous thrombosis.
- Our review highlights the need for increased vigilance for venous thromboembolism, with a low threshold for doppler ultrasound and CT in high-risk in-patient cohorts, where clinical features and D-dimer levels may not accurately reflect the occurrence of pulmonary thromboembolism.

## Introduction

COVID-19, a viral respiratory illness caused by the severe acute respiratory syndrome coronavirus 2, may predispose patients to thrombotic disease, both in the venous and arterial circulations, due to excessive inflammation, platelet activation, endothelial dysfunction, and stasis [1].

Screening for venous thromboembolism (VTE) in hospitalized COVID-19 patients is challenging. On the one hand, one should avoid a surge in upper and/or lower vascular ultrasound studies to avoid unnecessary prolonged direct exposure of staff to infected patients, avoid the risk of transmission of COVID-19 between patients via sonographers and avoid unnecessary wastage of personal protective equipment (PPE) [2]. On the other hand, the incidence of VTE in critically-ill COVID-19 patients is high and standard clinical symptoms associated with lower or upper extremity thrombus, such as swelling and pain, are often difficult to assess in intubated patients [3]. Therefore, timing, patient selection and VTE-scanning methods should be critically reassessed based on local availability of staff and PPE.

## Doppler ultrasound scan (DUS)

### On the general ward (non-ICU)

Several studies report a lower incidence of VTE in COVID-19 patients hospitalized on a medical ward (ranging between 1.6 and 22.5%) as compared to intensive care unit (ICU)-patients (25%–100%) [4, 5]. Lerardi et al*.* report a deep venous thrombosis (DVT) incidence of 1.6% among 60 non-ICU patients, routinely screened by doppler ultrasound [6]. In a prospective study of 156 non-ICU COVID-19 patients with D-dimer levels > 1000 ng/ml, compression ultrasound screening for asymptomatic DVT showed an incidence of 14.7% (only one proximal DVT) [7]; findings in line with another systematic screening for the diagnosis of deep vein thrombosis (DVT) by lower limb vein compression ultrasonography in 84 consecutive non-ICU patients despite uniform pharmacological thromboprophylaxis (incidence of 11.9%; 2.4% proximal DVT). Again, high D-dimer levels (> 3000 µg/L) were indicative of the presence of asymptomatic VTE [8]. In a retrospective cohort study of 71 non-ICU treated COVID-19 patients on anticoagulant thromboprophylaxis, who underwent systematic low limb venous duplex ultrasonography at hospital discharge or earlier if DVT was clinically suspected, 16 patients developed VTE (22.5%) and 7 PE (10%) [9].

### On the intensive care unit (ICU)

As previously mentioned, the incidence of DVT in critically-ill COVID-19 ICU patients is high, despite prophylactic anticoagulation strategies. A cross-sectional study in Wuhan, China in 48 ICU patients on low molecular weight heparin (LMWH) thromboprophylaxis detected lower extremity DVTs in 41 patients (85.4%), with 36 (75%) isolated distal DVTs and 5 (10.4%) proximal DVT [10]. In another retrospective study from Wuhan, all 88 ICU patients on LMWH thromboprophylaxis underwent compression ultrasonography. Here, the overall incidence of DVT was 46% (95% confidence interval [CI] 35–56%), comprising of proximal DVT in 9% (95% CI 3–15%) and distal DVT in 46% (95% CI 35–56%) [11]. Several other studies performed systematic DUS screening in COVID-19 ICU patients on prophylactical anticoagulation and report VTE incidences of 58.6% up to 100% [12, 13] or DVT incidences of 65% (at admission; and 79% 48 h after admission) [14], 36% [15], 37.3% [16] and 46.1% [3] respectively. Remarkably, Grandmaison et al*.* report a high VTE-incidence of 56% even in ICU patients receiving therapeutic anticoagulation [12]. A recent systematic review of 28 studies including a total of 2,928 patients with COVID-19 found that thrombotic complications were reported in 34% of ICU-managed patients, with DVT in 16.1% and PE in 12.6% of patients, despite anticoagulant thromboprophylaxis [17]. Studies adopting systematic screening for VTE with Duplex ultrasound reported a significantly higher incidence of venous thrombosis on the ICU compared to those relying on clinical suspicion (56.3% vs. 11.0%, p < 0.001) [17].

### Timing and patient selection for doppler ultrasound

There is no clear guidance on how screening for VTE in COVID-19 patients should be organized. Some centres perform routine DUS in every hospitalized patient [12], some centres only in critically-ill patients [6] and others only when VTE-symptoms are present. In a study of patients on 2 French ICUs, where DUS is performed as a standard of care, the overall VTE incidence was reported to be 69% [13]. As previously discussed, in their multi-centric retrospective study of 388 COVID-19 patients, Lodigiani et al*.* reported a VTE incidence of 27.6% in the ICU without systematic ultrasound screening [18]. This difference lends strong support to systematic screening by DUS for COVID-19 patients on ICU. In another cohort study, all hospitalized (non-ICU and ICU) COVID-19 patients were screened with DUS on a single day (320 patients) [12]. Patients confirmed to have VTE were switched to therapeutic anticoagulation and followed by clinical evaluation and repeated DUS of the index venous thrombosis. Under therapeutic anticoagulation, they did not observe any extension of thrombosis or new symptomatic events. Davis et al*.* state that an ultrasound in patients with clinical suspicion of lower or upper limb DVT should only be considered in patients with high bleeding score (VTE-BLEED score) [19], otherwise therapeutic anticoagulation should be started based on clinical findings [20] or based on the correlation with respiratory and/or laboratory parameters (fraction of inspired oxygen, P/F ratio, respiratory rate, heparin administration, D-dimer > 4000 ng/ml, IL-6, high ferritin or CRP), conditions mainly affecting patients on ICU [6]. Whilst some papers discourage routine screening for VTE by DUS on regular wards in asymptomatic or less sick patients [6], others select patients for screening based on the VTE-BLEED score [21], dependent on the D-dimer level (> 3 × upper limit of normal or > 4000 ng/mL) [2] or based on the (modified) Wells score. However, D-dimer is not so useful in the COVID-19 cohort due to frequent marked baseline levels observed in patients with COVID-19 sepsis [22]. Nevertheless, a negative Wells score or Wells score below 2 should be reassuring, negating the need for additional imaging [21].

### Doppler ultrasound scanning methods

Prolonged exposure of vascular sonographers to patients with COVID-19 should be carefully considered and balanced against the risks to staff and available resources, including staffing and PPE [2, 21]. Local scanning protocols should be re-evaluated to minimize the scanning time and thus exposure per patient. In one study, direct communication between physicians resulted in cancellation or deferral of 72% of requested examinations in COVID-19-positive patients. Preserving DUS screening for patients with D-dimer > 4000 ng/mL resulted in a significant (50%) decrease in the scan time (P < 0.0001) in comparison with conventional protocol [2].

A routine four limbs and neck compression duplex ultrasound should be sufficient to diagnose the majority of DVTs with minimal exposure time; when high clinical suspicion arises, a more focused scan at the site of the swelling can be obtained [12]. If available, wireless ultrasound probes, covered in single use transparent films should be used to minimise the risk of viral contamination [23].

## Computerised tomography (CT)

Patients with COVID-19 have been shown to have an increased risk of pulmonary thrombosis and CT is the optimal modality to assess this. CT studies have shown that pulmonary thromboembolism in COVID-19 involves mainly the segmental and sub-segmental arteries of segments affected by consolidation and may represent pulmonary artery thrombosis due to severe lung inflammation and hypercoagulability rather than thromboembolism. Pulmonary thrombosis or thromboembolism is best detected with CT pulmonary angiography (CTPA). Amongst patients with COVID-19, the yield of CTPA for pulmonary thromboembolism is around 38% [24, 25].

In the majority of patients with evidence of pulmonary thromboembolism, the most proximal PE was in a segmental artery in 70% of patients, and the most proximal PE was located in the main/lobar pulmonary artery in 17% of patients [26], with similar distributions reported by others [27].

A number of studies have assessed the usefulness of CT scanning to diagnose VTE in patients with COVID-19, both in hospitalised patients on the general wards and inpatients on the intensive care unit (ICU). Since the prevalence of PE relates to the severity of the COVID-19 infection, most studies report on the usefulness and yield of CTPA in patients on the ICU. However, data from a single-centre retrospective review of all CTPA studies in patients with suspected or confirmed COVID-19 showed that in 1477 patients, 214 CTPA scans were performed, of which 84% were requested outside of the critical care setting. The overall proportion of PE in patients with COVID-19 was 5.4%. Amongst patients with a Wells score of ≥ 4 (‘PE likely’), 25% had PE and 25% did not. D-dimer was higher in patients with PE than without PE (median 8000 ng/mL; interquartile range [IQR] 4665–8000 ng/mL vs. 2060 ng/mL, IQR 1210–4410 ng/mL, P < 0.001). In the ‘low probability’ group, D-dimer was higher (P < 0.001) in those with PE but had a limited role in excluding PE. Of all the patients with PE on CTPA, 66% were outside the ICU and nearly half of PE events were diagnosed on hospital admission [25]. This is surprising because it is known that the risk of VTE increases with the duration of hospitalisation. In a single-centre cross-sectional study, all 70 patients hospitalized for more than 5 days in with COVID-19 pneumonia and treated with standard anticoagulant thromboprophylaxis, underwent 2-point compressive ultrasound assessment of the leg vein system. Asymptomatic DVT was identified in 9 (13.6%) patients and CTPA detected PE in five patients [28].

### On the intensive care unit

Whilst in all-comers hospitalised with COVID-19, the prevalence of PE on CTCA was 5.4% [25, 29, 30], the yield of CTCA for PE ranges from 34 to 100% amongst patients on the ICU. In a retrospective review of 92 patients with ARDS on the ICU, 26 of the patients underwent CTPA and PE was detected in 16 patients, showing that CTPA had 62% yield for PE in this cohort. When present, PE was unilateral in 81% and bilateral in 19% of patients. The most proximal thrombus was localized in main (25%), lobar (12%) or segmental (63%) pulmonary artery. Most of the thrombi (81%) were located in a parenchymatous condensation. Only 19% of patients with PE on CT had lower limb DVT on Doppler ultrasound [27]. A couple of studies have reported on cohorts with COVID-19 on the ICU in whom routine CT scanning was performed. In an observational study of 39 consecutive mechanically-ventilated patients, of whom 51% received ECMO, all patients were scanned in a dedicated COVID-19 CT suite and PE was detected in 38.5% of patients [30]. In another single-centre study of 72 consecutive COVID-19 patients admitted to ICU with acquired respiratory distress syndrome, CT angiography of the thorax, abdomen and pelvis were performed on admission according to routine institutional protocol, with further imaging as clinically indicated. Some 58% of patients were diagnosed on CT to have thrombotic complications, comprising 47% of patients with pulmonary arterial thrombosis, 21% with peripheral venous thrombosis, and 7% with systemic arterial thromboses/end-organ embolic complications. In those with pulmonary arterial thromboses, 93% were identified incidentally on first screening CT with only 7% suspected clinically. Biomarkers of coagulation including D-dimer, fibrinogen level, activated partial thromboplastin time or of inflammation (white cell count, C-reactive protein), did not discriminate between patients with or without thrombotic complications [31].

### In patients receiving extra-corporeal membrane oxygenation (ECMO)

A retrospective observational analysis of 13 patients with ARDS requiring veno-venous ECMO, who all underwent CT, reported that 100% of the patients experienced venous thromboembolism, despite treatment with and close attention to anticoagulation. Ten patients had isolated cannula-associated DVT, two patients had isolated PE, and one patient had both cannula-associated DVT and PE. One patient had thrombotic occlusion of the centrifugal pump, and one had oxygenator thrombosis requiring circuit replacement [29]. Another report assessing the CT thorax of all 51 patients receiving ECMO with COVID-19 pneumonitis in a single centre showed that majority of patients had areas of ischaemia within consolidated lungs, almost half of these without subtending pulmonary artery thrombosis. Some 35% had macroscopic thrombosis and 26% had ischaemia without associated thrombus [32].

### Usefulness of CT

It has been suggested that D-dimer should be used as a guide to indicate the likelihood of PE when no clinical features of PE are present [33]. However, estimation of D- dimer levels for predicting thrombosis risk, whilst useful in patients presenting to hospital, is generally not helpful, given the significant baseline elevations in ICU-treated COVID-19 patients. The Report of the National Institute for Public Health of the Netherlands [33] recommends that in patients with COVID-19 in whom there is a high clinical suspicion for PE, and in whom the D-dimer level is elevated, CTPA should be considered. The D-dimer threshold used should follow locally used algorithms, that is, at least 500 µg/L with an age-adjusted threshold or 1000 µg/L or greater when no age criteria are present. In patients with a D-dimer < 1000 µg/L on admission but a significant increase during hospital stay to levels higher than 2000–4000 µg/L, imaging for DVT or PE should be considered, in particular if they develop features indicative of thrombosis such as venous congestion and/or thrombosis on chest CT scans, clotting of extracorporeal circuits, or deterioration in clinical condition such as hypoxia or hypotension.

In summary, CT is very useful modality to identify pulmonary thrombosis and embolism. The incidence of pulmonary thromboembolism increases with the severity of COVID-19 sepsis and so the yield of CTPA increases in the higher risk patients and in those on ICU, there should be a low threshold for requesting CTPA.

## Sublingual ultrasound

COVID-19 is associated not only with macrovascular, but also microvascular thrombosis. A couple of small, exploratory studies have assessed the presence of sublingual microcirculatory and skin perfusion alterations in COVID-19 pneumonia. In a small prospective observational study of 27 mechanically ventilated patients with ARDS secondary to COVID-19, sublingual microcirculation was assessed by hand-held video microscopy with subsequent software-assisted analysis of videos [34]. Capillary refill time was prolonged, total and perfused vascular density were high; and the proportion of perfused vessels, microvascular flow index and red blood cell velocity were reduced. The proportion of perfused vessels was inversely correlated with total vascular density. These findings were indicative of microvascular thrombosis. In another small observational cohort study of 13 patients on the ICU, without clinical features of cardiogenic shock, in vivo evaluation of the sublingual microcirculation was performed using a handheld video capillaroscopy system with imaging analysed using the specialised software [35]. There was evidence of microvascular thrombosis in 11 patients (85%). Four patients (31%) exhibited complete stagnated capillaries and in 5 (38%) patients imaging indicated acute thromboembolic occlusion.

## Discussion

**Fig. 1:**
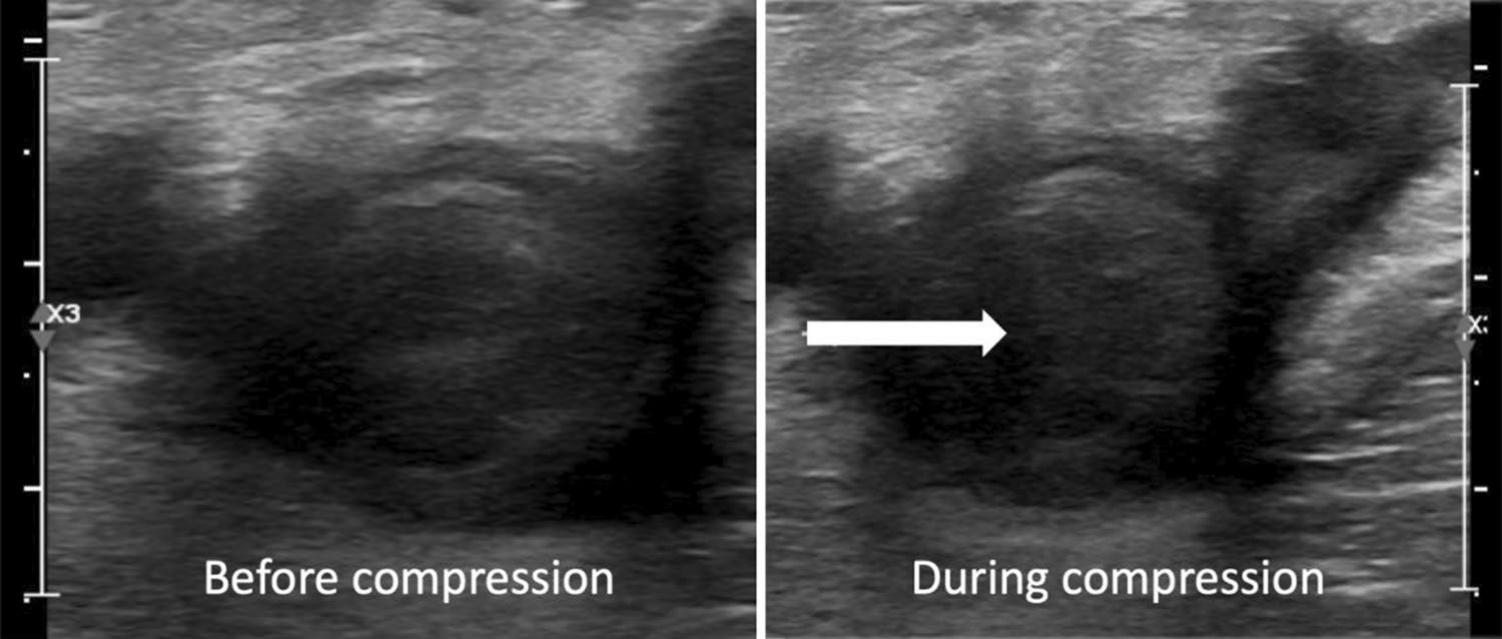
Venous compression ultrasound in a 43-year-old male COVID-19 patient, requiring mechanical ventilation on the intensive care unit (left panel: before compression, right panel: during compression). The ultrasound reveals a large, semi-occlusive venous clot in the right common iliac vein, indicated by the white arrow

Pulmonary and venous thrombosis occur frequently in patients with COVID-19 and pulmonary thrombosis is frequently non-embolic. The prevalence increases in ICU patients and is very high in patients on ECMO. In patients outside of the ICU setting, D-dimer elevation on presentation or marked increase from baseline should alert the need for doppler ultrasound scan of the lower limbs. In the ICU setting, consideration should be given to routine screening with doppler ultrasound, given the high prevalence of thrombosis in this cohort in the setting of standard anticoagulant thromboprophylaxis, if the diagnosis would alter management. Pulmonary thromboembolism usually affects distal vasculature. Screening with CTCA is not justified in patients on the general wards, unless there are clinical features and/or marked elevations in markers of COVID-19-associated coagulopathy. However, the risk of PE in ICU patients is very high, and especially in patients on ECMO, where studies that employed routine screening have uncovered up to 100% incidence of pulmonary thrombosis despite standard anticoagulant thromboprophylaxis. This highlights the need for increased dose thromboprophylaxis in high-risk patients, if possible, and increased vigilance, with a low threshold for requesting CTPA and understanding that lack of lower limb thrombosis on ultrasound does not exclude pulmonary venous thrombosis. The logistics of CT scanning all ICU patients with COVID-19 in most centres without a dedicated co-located CT scanner makes this unfeasible, both in terms of infection risk and also the risk of transporting such critically ill patients across the hospital. Therefore, in patients with low bleeding risk and high clinical suspicion of VTE, the initiation of therapeutic anticoagulation without confirmation of VTE by imaging should be considered. However, since clinical features and D-dimer levels may not accurately reflect the occurrence of pulmonary thromboembolism, more data are needed to identify an optimal diagnostic pathway in patients with COVID-19 (Fig. 1).

## Abbreviations

COVID-19: Coronavirus 19
CT: Computerised tomography
CTPA: Computerised tomography pulmonary angiogram
DUS: Doppler ultrasound scan
DVT: Deep venous thrombosis
ICU: Intensive care unit
LMWH: Low molecular weight heparin
PE: Pulmonary embolism
PPE: Personal protective equipment
VTE: Venous thromboembolism
